# Poly[(μ_2_-2-amino­pyrimidine-κ^2^ *N* ^1^:*N* ^3^)di-μ_2_-chlorido-mercury(II)]

**DOI:** 10.1107/S1600536812008793

**Published:** 2012-03-07

**Authors:** Hossein Eshtiagh-Hosseini, Zakieh Yousefi, Agnieszka Janiak

**Affiliations:** a Department of Chemistry, Ferdowsi University of Mashhad, 917791436 Mashhad, Iran; b Faculty of Chemistry, Adam Mickiewicz University, Grunwaldzka 6, 60-780 Poznań, Poland

## Abstract

The title compound, [HgCl_2_(C_4_H_5_N_3_)]_*n*_, features a two-dimensional network parallel to (001) that is based on an Hg^II^ atom octahedrally coordinated by four μ_2_-Cl atoms and two μ_2_-2-amino­pyrimidine (apym) ligands in *trans* positions, yielding a distorted HgCl_4_N_2_ octa­hedron. The coordination network can be described as an uninodal 4-connected net with the sql topology. The Hg^II^ ion lies on a site of -1 symmetry and the apym ligand lies on sites of *m* symmetry with the mirror plane perpendicular to the pyrimidine plane and passing through the NH_2_ group N atom. This polymeric structure is stabilized by N—H⋯Cl hydrogen bonds and columnar π–π stacking of pyrimidine rings, with a centroid–centroid distance of 3.832 (2) Å.

## Related literature

For pyridine complexes of mercury(II) halides see: Hu *et al.* (2007 ▶). For mercury(II) coordination polymers, see: Mahmoudi & Morsali (2009 ▶). For the same topological type of two-dimensional coordination networks, see: Nockemann & Meyer (2004 ▶); Xie & Wu (2007 ▶). For topological analysis, see: Blatov (2006 ▶). For an isotypic Cd^II^ complex, see: Salinas-Castillo *et al.* (2011 ▶). For our previous work on structures with an apym ligand, see: Eshtiagh-Hosseini *et al.* (2009 ▶, 2010 ▶, 2011 ▶).
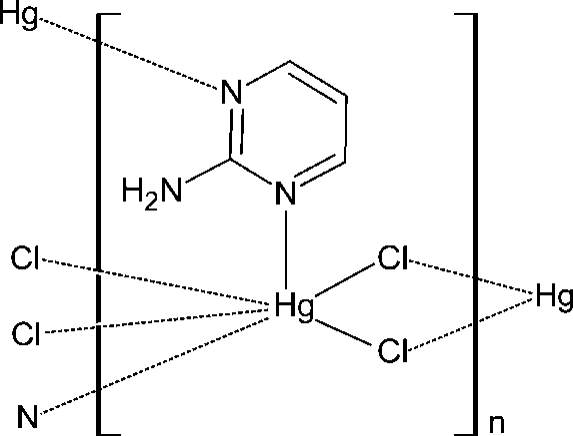


## Experimental

### 

#### Crystal data

[HgCl_2_(C_4_H_5_N_3_)]
*M* *_r_* = 366.60
Monoclinic, 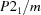

*a* = 3.8317 (1) Å
*b* = 14.1366 (3) Å
*c* = 7.0773 (2) Å
β = 96.814 (2)°
*V* = 380.65 (2) Å^3^
*Z* = 2
Mo *K*α radiation
μ = 20.84 mm^−1^
*T* = 294 K
0.45 × 0.04 × 0.02 mm

#### Data collection

Oxford Diffraction Xcalibur E diffractometer
Absorption correction: multi-scan (*CrysAlis PRO*; Agilent, 2011 ▶) *T*_min_ = 0.160, *T*_max_ = 1.000
9437 measured reflections
992 independent reflections
867 reflections with *I* > 2σ(*I*)
*R*_int_ = 0.033

#### Refinement

*R*[*F* ^2^ > 2σ(*F* ^2^)] = 0.020
*wR*(*F* ^2^) = 0.049
*S* = 1.12
992 reflections
52 parameters
H-atom parameters constrained
Δρ_max_ = 0.84 e Å^−3^
Δρ_min_ = −1.01 e Å^−3^

### 

Data collection: *CrysAlis PRO* (Agilent, 2011 ▶); cell refinement: *CrysAlis PRO*; data reduction: *CrysAlis PRO*; program(s) used to solve structure: *SHELXS97* (Sheldrick, 2008 ▶); program(s) used to refine structure: *SHELXL97* (Sheldrick, 2008 ▶); molecular graphics: *Mercury* (Macrae *et al.*, 2008 ▶); software used to prepare material for publication: *publCIF* (Westrip, 2010 ▶).

## Supplementary Material

Crystal structure: contains datablock(s) I, global. DOI: 10.1107/S1600536812008793/gk2458sup1.cif

Structure factors: contains datablock(s) I. DOI: 10.1107/S1600536812008793/gk2458Isup2.hkl

Additional supplementary materials:  crystallographic information; 3D view; checkCIF report

## Figures and Tables

**Table:** 

Hg1—Cl1	2.3987 (8)
Hg1—N2	2.618 (3)
Hg1—Cl1^i^	2.9881 (9)

**Table:** 

Cl1—Hg1—N2^ii^	91.45 (7)
Cl1—Hg1—N2	88.55 (7)
N2^ii^—Hg1—N2	180.0
N2—Hg1—Cl1^iii^	92.95 (7)
Cl1—Hg1—Cl1^i^	90.00 (3)
N2—Hg1—Cl1^i^	87.05 (7)

Symmetry codes: (i) 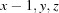; (ii) 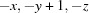; (iii) 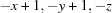.

**Table 2:** Hydrogen-bond geometry (Å, °)

*D*—H⋯*A*	*D*—H	H⋯*A*	*D*⋯*A*	*D*—H⋯*A*
N1—H1⋯Cl1^iii^	0.96	2.41	3.363 (3)	173

Symmetry code: (iii) 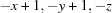.

**Fig. 1.**
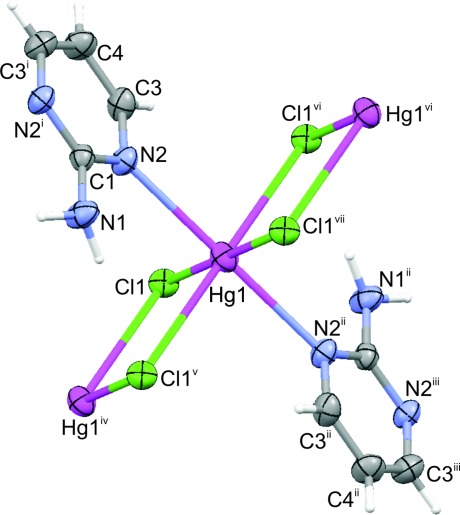
ORTEP view of coordination sphere of mercury (II). Symmetry code: (i) x, 1.5 - y, z; (ii) -x, 1 - y, -z; (iii) -x, -0.5 + y, -z; (iv) 1 + x, y, z; (v) 1 - x, 1 - y, -z; (vi) -1 + x, y, z; (vii) -x, 1 - y, -z. Displacement ellipsoids are given at the 50% probability level.

**Fig. 2.**
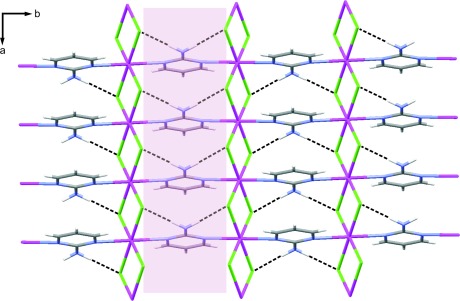
Representation of two-dimensional coordination polymer. Dashed lines denote intermolecular N— H···Cl hydrogen bonds. The columnar π–π stacking interactions are highlighted in pink.

## supplementary crystallographic information

### Comment

Mercury with its d^10^ electronic configuration exhibits a wide range of geometry in coordination sphere giving rise to a variety of topological types of one-dimensional, two-dimensional and three-dimensional polymers (Mahmoudi & Morsali, 2009).

In this contribution, we have synthesized and characterized a two-dimensional framework containing [Hg(apym)Cl_2_] unit in which Hg^II^ ion is six coordinated *via* four chloride anions and a two apym molecules (Fig. 1). Hg^II^ ion exhibits slightly distorted octahedral coordination geometry for which the maximum deviation of twelve octahedral angles from an ideal 90° for *cis* angles is 0.82 (7)°.

In the crystal structure, Hg^II^ ion lies on an inversion centre and the apym molecule lies on a special position of *m* site symmetry with mirror plane passing through an amino nitrogen. Hg^II^ ions are connected to each other by the bridging chloride ions in [100] direction; the seperation between the two bridged Hg^II^ ions is 3.8317 (1) Å, and is shorter than that in [Hg(µ_2_-Cl)_2_(C_7_H_9_N)_2_(µ_2_HgCl)_3_]_∞_ [3.9960 (9) Å, Hu *et al.*, 2007]. The four-membered Hg_2_Cl_2_ ring is planar and contains pairs of long and short Hg— Cl bonds [Hg1— Cl1 2.9881 (9) Å & Hg1— Cl1 2.3987 (8) Å]. The apym molecule acts as a bidentate ligand that links two neighbouring Hg^II^ ions in the crystallographic *b* direction with seperation Hg1···Hg1 distance of 7.0683 (3) Å, in consequence leading to formation of two-dimensional infinite framework with grid size of 7.068×3.832 Å^2^ (Fig. 2). The topological type of this layer arrangement is sql {4^4^.6^2^}(Blatov, 2006). Similar two-dimensional neutral polymer consisting of mercury(II) ions bridged by both pyrazine and bromide ligands has been reported by Mahmoudi & Morsali (2009). The title compound is isostructural with its Cd analogoue, reported by Salinas-Castillo *et al.* (2011).

It is noteworthy that in our previous works, apym either played a role of a counter ion for an anionic network or acted as an uncharged monodentate ligand ( Eshtiagh-Hosseini *et al.*, 2009, 2010, 2011).

Another point of interest is the existance of N— H···Cl hydrogen bonds as well as columnar π–π interactions between pyrimidine rings of apym ligands which are arranged into stacks propagating in the *a* direction (see Fig. 2) with the perpendicular separation of 3.509 (2) Å and the centroid-to-centroid distance of 3.832 (2) Å.

### Experimental

To a solution of HgCl_2_ (0.050 g, 0.2 mmol) in 10 ml of MeOH was added dropwise a solution of pyridine-2,5-dicarboxylic acid (0.018 g, 0.1 mmol) in 10 ml of MeOH in the reflux condition. After 15 min, 2-aminopyrimidine (0.020 g, 0.2 mmol) was added as solid form, and the resultant solution stirred and refluxed for 12 h. After cooling the solution, a colourless needle-like crystals were obtained (yield: 70%).

### Refinement

H atoms bound to C atoms were placed in their idealized positions and were refined as riding on their parent atoms with C—H distance of 0.93 Å. The symmetry independent amine H-atom was first found in a difference Fourier map and then refined using a riding model with *U*_iso_═ 1.2*U*_iso_(N). The highest peak in the final electron density difference map is located 1.07 Å from Hg1 atom.

### Crystal data

**Table:**

[HgCl_2_(C_4_H_5_N_3_)]	*F*(000) = 328
*M**_r_* = 366.60	*D*_x_ = 3.198 Mg m^−^^3^
Monoclinic, *P*2_1_/*m*	Mo *K*α radiation, λ = 0.71073 Å
Hall symbol: -P 2yb	Cell parameters from 3794 reflections
*a* = 3.8317 (1) Å	θ = 2.9–29.0°
*b* = 14.1366 (3) Å	µ = 20.84 mm^−^^1^
*c* = 7.0773 (2) Å	*T* = 294 K
β = 96.814 (2)°	Needle, colourless
*V* = 380.65 (2) Å^3^	0.45 × 0.04 × 0.02 mm
*Z* = 2	

### Data collection

**Table:**

Oxford Diffraction Xcalibur E diffractometer	992 independent reflections
Radiation source: Enhance (Mo) X-ray Source	867 reflections with *I* > 2σ(*I*)
Graphite monochromator	*R*_int_ = 0.033
Detector resolution: 16.1544 pixels mm^-1^	θ_max_ = 29.0°, θ_min_ = 2.9°
ω scans	*h* = −5→4
Absorption correction: multi-scan (*CrysAlis PRO*; Agilent, 2011)	*k* = −18→19
*T*_min_ = 0.160, *T*_max_ = 1.000	*l* = −9→9
9437 measured reflections	

### Refinement

**Table:**

Refinement on *F*^2^	Primary atom site location: structure-invariant direct methods
Least-squares matrix: full	Secondary atom site location: difference Fourier map
*R*[*F*^2^ > 2σ(*F*^2^)] = 0.020	Hydrogen site location: inferred from neighbouring sites
*wR*(*F*^2^) = 0.049	H-atom parameters constrained
*S* = 1.12	*w* = 1/[σ^2^(*F*_o_^2^) + (0.0245*P*)^2^ + 0.3601*P*] where *P* = (*F*_o_^2^ + 2*F*_c_^2^)/3
992 reflections	(Δ/σ)_max_ < 0.001
52 parameters	Δρ_max_ = 0.84 e Å^−^^3^
0 restraints	Δρ_min_ = −1.01 e Å^−^^3^

### Special details

**Table:**

Geometry. All e.s.d.'s (except the e.s.d. in the dihedral angle between two l.s. planes) are estimated using the full covariance matrix. The cell e.s.d.'s are taken into account individually in the estimation of e.s.d.'s in distances, angles and torsion angles; correlations between e.s.d.'s in cell parameters are only used when they are defined by crystal symmetry. An approximate (isotropic) treatment of cell e.s.d.'s is used for estimating e.s.d.'s involving l.s. planes.
Refinement. Refinement of *F*^2^ against ALL reflections. The weighted *R*-factor *wR* and goodness of fit *S* are based on *F*^2^, conventional *R*-factors *R* are based on *F*, with *F* set to zero for negative *F*^2^. The threshold expression of *F*^2^ > σ(*F*^2^) is used only for calculating *R*-factors(gt) *etc*. and is not relevant to the choice of reflections for refinement. *R*-factors based on *F*^2^ are statistically about twice as large as those based on *F*, and *R*- factors based on ALL data will be even larger.

### Fractional atomic coordinates and isotropic or equivalent isotropic displacement parameters (Å^2^)

**Table:**

	*x*	*y*	*z*	*U*_iso_*/*U*_eq_	
Hg1	0.0000	0.5000	0.0000	0.03124 (9)	
Cl1	0.4453 (2)	0.44666 (6)	0.24361 (12)	0.02963 (19)	
C1	0.0775 (12)	0.7500	0.0879 (7)	0.0249 (10)	
N1	0.1926 (13)	0.7500	−0.0838 (7)	0.0355 (10)	
H1	0.2972	0.6915	−0.1176	0.043*	
N2	0.0202 (8)	0.6654 (2)	0.1677 (4)	0.0277 (6)	
C4	−0.1355 (15)	0.7500	0.4342 (8)	0.0353 (12)	
H4	−0.2054	0.7500	0.5556	0.042*	
C3	−0.0821 (10)	0.6677 (3)	0.3413 (5)	0.0327 (8)	
H3	−0.1185	0.6107	0.4016	0.039*	

### Atomic displacement parameters (Å^2^)

**Table:**

	*U*^11^	*U*^22^	*U*^33^	*U*^12^	*U*^13^	*U*^23^
Hg1	0.02661 (13)	0.03241 (14)	0.03432 (14)	0.00086 (7)	0.00197 (9)	0.00342 (8)
Cl1	0.0305 (4)	0.0297 (4)	0.0287 (4)	0.0022 (3)	0.0036 (3)	0.0019 (4)
C1	0.023 (2)	0.023 (2)	0.028 (3)	0.000	0.000 (2)	0.000
N1	0.051 (3)	0.022 (2)	0.037 (3)	0.000	0.017 (2)	0.000
N2	0.0347 (16)	0.0219 (15)	0.0261 (15)	−0.0005 (12)	0.0014 (12)	0.0007 (12)
C4	0.043 (3)	0.040 (3)	0.024 (3)	0.000	0.007 (2)	0.000
C3	0.040 (2)	0.029 (2)	0.0280 (19)	−0.0055 (16)	0.0025 (16)	0.0036 (16)

### Geometric parameters (Å, º)

**Table:**

Hg1—Cl1^i^	2.3987 (8)	C1—N2	1.352 (4)
Hg1—Cl1	2.3987 (8)	C1—N2^v^	1.352 (4)
Hg1—N2^i^	2.618 (3)	N1—H1	0.9618
Hg1—N2	2.618 (3)	N2—C3	1.334 (5)
Hg1—Cl1^ii^	2.9881 (9)	C4—C3	1.364 (5)
Hg1—Cl1^iii^	2.9881 (9)	C4—C3^v^	1.364 (5)
Cl1—Hg1^iv^	2.9881 (9)	C4—H4	0.9300
C1—N1	1.340 (6)	C3—H3	0.9300
			
Cl1^i^—Hg1—Cl1	180.00 (3)	Hg1—Cl1—Hg1^iv^	90.00 (3)
Cl1^i^—Hg1—N2^i^	88.55 (7)	N1—C1—N2	117.7 (2)
Cl1—Hg1—N2^i^	91.45 (7)	N1—C1—N2^v^	117.7 (2)
Cl1^i^—Hg1—N2	91.45 (7)	N2—C1—N2^v^	124.5 (5)
Cl1—Hg1—N2	88.55 (7)	C1—N1—H1	114.7
N2^i^—Hg1—N2	180.0	C3—N2—C1	116.3 (4)
Cl1^i^—Hg1—Cl1^ii^	90.00 (3)	C3—N2—Hg1	116.3 (2)
Cl1—Hg1—Cl1^ii^	90.00 (3)	C1—N2—Hg1	126.8 (3)
N2^i^—Hg1—Cl1^ii^	87.05 (7)	C3—C4—C3^v^	117.1 (5)
N2—Hg1—Cl1^ii^	92.95 (7)	C3—C4—H4	121.4
Cl1^i^—Hg1—Cl1^iii^	90.00 (3)	C3^v^—C4—H4	121.4
Cl1—Hg1—Cl1^iii^	90.00 (3)	N2—C3—C4	122.9 (4)
N2^i^—Hg1—Cl1^iii^	92.95 (7)	N2—C3—H3	118.6
N2—Hg1—Cl1^iii^	87.05 (7)	C4—C3—H3	118.6
Cl1^ii^—Hg1—Cl1^iii^	180.00 (3)		

Symmetry codes: (i) −*x*, −*y*+1, −*z*; (ii) −*x*+1, −*y*+1, −*z*; (iii) *x*−1, *y*, *z*; (iv) *x*+1, *y*, *z*; (v) *x*, −*y*+3/2, *z*.

### Hydrogen-bond geometry (Å, º)

**Table:**

*D*—H···*A*	*D*—H	H···*A*	*D*···*A*	*D*—H···*A*
N1—H1···Cl1^ii^	0.96	2.41	3.363 (3)	173

Symmetry code: (ii) −*x*+1, −*y*+1, −*z*.
